# Epigenetic and metabolic reprogramming in inflammatory bowel diseases: diagnostic and prognostic biomarkers in colorectal cancer

**DOI:** 10.1186/s12935-023-03117-z

**Published:** 2023-11-07

**Authors:** Zeinab Deris Zayeri, Abazar Parsi, Saeid Shahrabi, Masoud Kargar, Nader Davari, Najmaldin Saki

**Affiliations:** 1 Golestan Hospital Clinical Research Development Unit, Ahvaz Jundishapur University of Medical Sciences, Ahvaz, Iran; 2 Alimentary Tract Research Center, Clinical Sciences Research Inistitute, Ahvaz Jundishapur University of Medical Sciences, Ahvaz, Iran; 3 Department of Biochemistry and Hematology, Faculty of Medicine, Semnan University of Medical Sciences, Semnan, Iran; 4 Thalassemia and Hemoglobinopathy Research Center, Health Research Institute, Ahvaz Jundishapur University of Medical Sciences, Ahvaz, Iran

**Keywords:** Inflammatory bowel disease, Colorectal cancer, Transition, Biomarkers, Epigenetics, Metabolism

## Abstract

**Background and aim:**

"Inflammatory bowel disease" (IBD) is a chronic, relapsing inflammatory disease of the intestinal tract that typically begins at a young age and might transit to colorectal cancer (CRC). In this manuscript, we discussed the epigenetic and metabolic change to present a extensive view of IBDs transition to CRC. This study discusses the possible biomarkers for evaluating the condition of IBDs patients, especially before the transition to CRC.

**Research approach:**

We searched “PubMed” and “Google Scholar” using the keywords from 2000 to 2022.

**Discussion:**

In this manuscript, interesting titles associated with IBD and CRC are discussed to present a broad view regarding the epigenetic and metabolic reprogramming and the biomarkers.

**Conclusion:**

Epigenetics can be the main reason in IBD transition to CRC, and Hypermethylation of several genes, such as *VIM, OSM4, SEPT9, GATA4 and GATA5, NDRG4, BMP3, ITGA4 and* plus hypomethylation of *LINE1* can be used in IBD and CRC management. Epigenetic, metabolisms and microbiome-derived biomarkers, such as Linoleic acid and 12 hydroxy 8,10-octadecadienoic acid, Serum M2-pyruvate kinase and Six metabolic genes (*NAT2, XDH, GPX3, AKR1C4, SPHK* and *ADCY5*) expression are valuable biomarkers for early detection and transition to CRC condition. Some miRs, such as *miR-31, miR-139-5p, miR -155, miR-17, miR-223, miR-370-3p, miR-31, miR -106a, miR -135b and miR-320* can be used as biomarkers to estimate IBD transition to CRC condition.

## Highlights

1. Inflammatory signals, cytokines and immune cell interaction lead to epigenetic reprogramming in IBD.
2. The imprinting profile of IGF2, NOD2, ATG16L1 and IRGM is considerable in IBD.
3. Microbiota plays an essential role in IBD transition to CRC via NF-kB and STAT3 signaling pathways.
4. TLR4 activates β-catenin via PI3K then it interacts with Wnt pathway and leads to a metabolic and neoplastic reprograming in IBD.

## Introduction

Chronic inflammatory disease of the gastrointestinal tract called inflammatory bowel disease (IBD) is a multi-factorial disease caused by the interplay of hereditary and environmental factors. Although more than 160 loci have been linked to IBD, the function of many of them remains unknown [1]. The developmental origins of IBD, including Crohn's disease (CD) and ulcerative colitis (UC), which are connected to early-onset CRC, have been linked to epigenetic and microbiome modifications. Intercalating age-specific DNA methylation and microbiome changes have been reported in various studies, and they may be significantly relevant to the developmental causes of IBD and CRC [2]. Ng et al. conducted a very precious systematic review regarding the prevalence of IBD on 147 studies around the world. The conclusion of this report mentioned that in Europe (UC 505 per 100 000 in Norway; CD 322 per 100 000 in Germany) and North America (UC 286 per 100 000 in the USA; CD 319 per 100 000 in Canada), were reported as the highest reported prevalence. In North America, Oceania, and many European nations, the prevalence of IBD exceeded 0.3 percent [3]. CD is a transmural but frequently segmental inflammation of the gut wall that can develop anywhere in the gut, from the mouth to the anus. Fistula or fibrosis development may be triggered by inflammation in CD. Up to 80% of patients are currently necessitated to undergo at least one surgical resection of an intestinal segment, despite recent advancements in medicinal therapy options [4]. In contrast, UC only affects the large bowel and always begins with the rectum displaying the maximum level of activity. Up to 10 to 15% of patients may require a colectomy since it may be characterized by a high rate of bloody bowel movements per day and significantly decreases the quality of life [5]. Research on IBD pathophysiology and variables causing disease outbreaks is currently considerable due to the associated substantial morbidity and—at least for many people with more severe disease—unsatisfactory treatment choices. IBD has become a "prototype disease group" for chronic auto inflammatory diseases with a polygenic basis and significant complex environmental factors. Environmental factors must be relevant to both the etiology of the disease and the flare of the disease [6]. Over hundreds or thousands of years, the genetic risk factors have not changed, but the incidence and prevalence of diseases remain increasing. IBD was almost nonexistent as a disease until a century ago, However, it affects millions of people, including non-Caucasians, among whom the disease had previously been incredibly rare nowadays, and this is obviously a consequence of the environmental factors [7]. Microbiome aberration (dysbiosis), immunological dysregulation, and prenatal, perinatal, and pediatric environmental effects all seem to play significant roles in the onset, progression, and maintenance of IBD. In highly industrialized countries, the frequency of combined genetic and epigenetic susceptibility to UC and CD is estimated to be as high as 2% and about 1%, respectively [8]. Unlike the hereditary syndromes of familial adenomatous polyposis (FAP) and hereditary nonpolyposis colorectal cancer (HNPCC) that have a well-defined genetic etiology, the transition condition of IBD to CRC seems to be related to the long-term inflammation and inflammatory responses which reveals the importance of epigenetic in IBD. The most important carcinogenic pathways in CRC development seem to be chromosomal instability, microsatellite instability, and hypermethylation, which are noticeable in inflamed colonic mucosa before histological events so this fact highlights the importance of molecular pathways in early diagnosis [9]. Epigenomic regulation, such as DNA methylation, histone modification, and non-coding RNA, play important roles in the normal development and function of an organism and epigenetic alterations play a critical role in complex diseases, such as IBD, cancer, obesity, and type 2 diabetes (T2D) [10, 11]. Aerobic glycolysis is the main mechanism for glucose metabolism in cancer cells, and metabolic changes in cancer and metastasis and the upregulation of glycolysis have been reported in many.

primary and metastatic cancers [12]. It is unknown if cell metabolism has been altered throughout the transition from IBD to colorectal cancer (CRC). Cancer cells ferment a large portion of their glucose supply into lactate regardless of the availability of oxygen. Ventham et al. conducted a study with a mouse colitis model produced by dextran sulfate sodium (DSS), and discovered that inflammation increased the expression of essential glycolytic enzymes by activating the STAT3/c-Myc signaling pathway. It's interesting to note that key metabolic enzymes consistently showed enhanced expression during the whole phase of chronic inflammation, demonstrating that the metabolic reprogramming was brought on by a long-lasting inflammatory signal. It is unknown if cell metabolism has been altered throughout the transition from IBD to CRC [13].

This manuscript is focused on recent developments in our understanding of how intestinal microbiota, DNA methylation, micro-RNAs, histone, and metabolism changes interact to IBD transition to CRC. Finally, we discuss the clinical and diagnostic implications, viewpoints, and non-invasive biomarkers surrounding the epigenetic and metabolic reprogramming in IBD.

### Epigenetic reprogramming in IBD can be an alarm of vulnerability

Epigenetics is an important factor in cancer development and it’s defined as heritable phenotypic changes brought on by factors other than the DNA sequence. Histone modification (including acetylation and methylation), DNA methylation and small noncoding RNAs (sncRNAs), such as micro RNAs (miRNAs), have also recently been discovered to function as epigenetic factors [14]. Epigenetic alterations are likely to play critical roles in various diseases, such as autoimmune disease [15], Alzheimer’s disease [16], mental disease [17] and cancer [18]. Inheritable epigenetic markers have been suggested to play a role in the pathogenesis of IBD more than ten years ago [19]. Epigenetic changes have fundamental characteristics which are inheritable and impact the phenotype. They can be passed down across subsequent cell divisions based on their heritable nature and, have the power to either directly or indirectly change the transcriptional status of the underlying DNA sequence. The ability of epigenetic modifications to be fully reversed, allowing for their erasure and subsequent reestablishment upon passage through the parental germlines, may be the most important trait of all. However, incomplete "resetting" of the epigenome can be the major reason for appearing some undesirable phenotypes [20]. Epigenetics may be the major reason in determining IBD outcome of specific malignancy like CRC, and there is a noticeable link between epigenetic, cell division and cancer [21]. Inflammatory signals, cytokines, immune cells interaction can change the epigenetics in IBD and lead to epigenetic reprogramming [22]. p53, DNA mismatch repair genes, and even DNA base excision-repair genes are examples of important genes implicated in carcinogenic pathways that can be impacted by reactive oxygen and nitrogen species produced by inflammatory cells [23]. Tumor-suppressor genes, including KLF6, TP53, APC, K-RAS and DCC have been reported to be inactivated in patients with IBD-associated cancer, for instance. The tumor-suppressor genes are silenced in collaboration with abnormal promoter methylation and without coding region abnormalities and mutations [24]. Interestingly, most of the epigenetic changes are completely reversible, they can be removed and then reinstated during mitosis and after passing through the parental germlines during meiosis. The most well-known inheritable epigenetic alterations include DNA methylation, histone posttranslational modifications and nucleosome positioning [25]. Chromatin remodeling complexes, the lauded polycomb group proteins, and micro RNAs (miRNAs), are recently considered inheritable epigenetic alterations. A variety of biological processes, such as gene transcription, DNA–protein interactions, protein translation and silencing of endogenous retrotransposons, are coordinated by epigenetic changes and their associated modifiers that influence important processes like growth, differentiation, immunity, X chromosome inactivation and genomic imprinting [26, 27]. Epigenetic alterations plasticity and reversibility nature also increase the cell vulnerability and make the cells susceptible to environmental factors, such as infection, inflammation, Oxidative Redox Stress (ROS) and chemical and pharmaceutical agents. Epigenetic changes have the potential to mediate the gene-environment interactions that underlie multifactorial diseases such as IBD [28]. When the epigenetic reprogramming starts and which factors trigger it, is an interesting discussion that we preferred to discuss in the next subtitles.

#### Change in gene imprinting in IBD and CRC

**Fig. 1:**
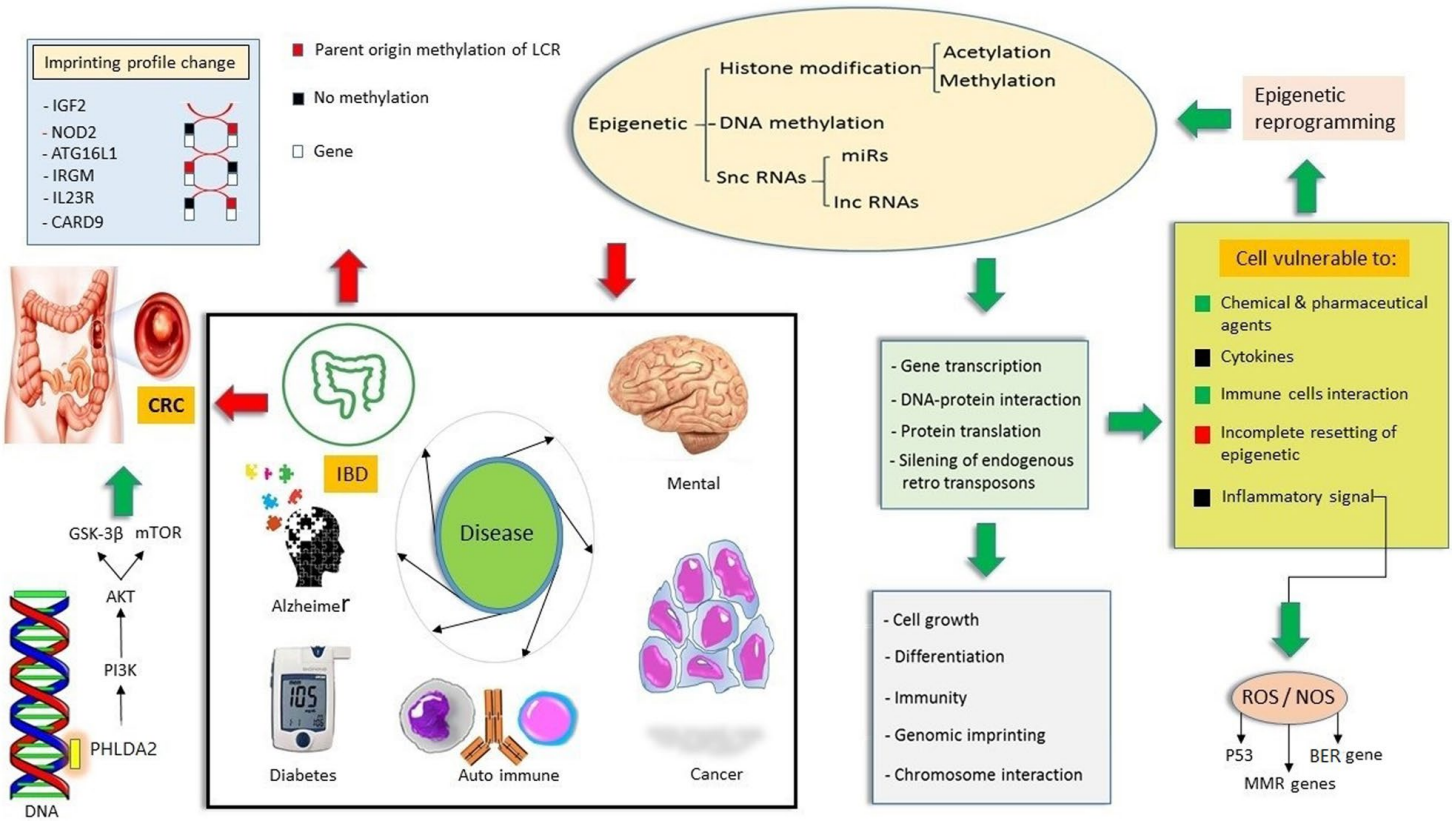
Epigenetic plays critical role in multi-factorial disease. Epigenetic changes affect gene transcription, DNA–Protein interaction, Protein translation, and silencing of endogenous retro transposons. Consequently, important functions, such as cell growth, differentiation, immunity, genomic imprinting, and chromosome interaction change and make the cell vulnerable to inflammatory signals, cytokines, and other agents and changes. These cells start epigenetic reprogramming in response to the new environmental changes. IGF2: Insulin-like growth factor II, NOD2: Nucleotide Binding Oligomerization Domain Containing 2, ATG16L1: Autophagy-Related 16-Like 1, IRGM: Immunity-related GTPase family M protein, IL23R: Interleukin 23 Receptor, CARD9: Caspase Recruitment Domain Family Member 9, PHLDA2: Pleckstrin homology like domain family A member 2, CRC: Colorectal cancer, IBD: Inflammatory bowel disease, ROS: Oxidative Redox Stress, NOS: Nitric oxide synthase, MMR, DNA: DNA mismatch repair, BER: base excision-repair

Recent studies of parent-of-origin effects on the transmission of familial IBD considered imprinting in several genes and the results suggested that the imprinting profile changed in several genes, such as *NOD2, ATG16L1, IRGM *[29]*, IL23R, CARD9, RNF186* and *PRDM1* [30, 31]. How the etiology of complex disorders might be influenced by genomic imprinting and the influence of genomic imprinting on immune functions and inflammatory disorders are still unknown [32]. Imprinted genes are extremely vulnerable to "loss of imprinting," a sort of dysregulation that can activate the normally silent allele. Imprinted gene products are extremely sensitive to dosage variations, therefore their altered expression as a result of loss of imprinting can cause various disease such as cancer [33]. Imprinted genes are expressed from a single parental allele and are essential for development, metabolism and neural function. This expression at imprint control region (ICRs) is regulated by parent-of-origin-dependent CpG methylation. These methylation markers are common to all cell types because ICRs are created earlier than tissue differentiation. As a result, they are interesting for analyzing the developmental origins of adult diseases [34]. Loss of imprinting (LOI) in genes such as insulin-like growth factor II (IGF2) gene was reported to increase the risk of CRC via increasing CRC stem cells pluripotency by promoting tumor autophagy [35]. Single cell RNA sequencing data analysis suggested that Loss of IGF2 Imprinting can be used as a potential biomarker to estimate the risk of CRC [36]. Mice study confirmed that mice with LOI in IGF2 gene present twice risk of intestinal tumors development which can be a valuable confirmation of the impact of epigenetic in cancer development [37]. pleckstrin homology like domain family A member 2 (PHLDA2) gene is a maternally imprinted gene that is highly imprinting in CRC. Downregulation of PHLDA2 suppresses tumor development via PI3K/AKT/mTOR and PI3K/AKT/GSK-3β signaling pathways [38] Fig. 1. Table 1 discuss several genes that were studied for imprinting alteration in IBD and CRC CRC-based studies.

**Table 1:** Genes showed imprinting alteration in IBD and CRC-based studies

Gene	Location	Study	Function	Result	Refernces
*NOD2 (CARD15) (IBD1)*	*Chr 16*	*Nod2*^−/−^ mice	Activates NF-kB, Controls inflammatory and immune responses	The long-lasting effects of neonatal antibiotic exposure on the microbiota and mucosal immunity may be the reason why NOD2 increases the risk of intestinal inflammation	[39]
*ATG16L1*	Chr 2	Transcriptomic study of UC mice model, colitis models, flora mice	Regulated the expression of lysozyme, changes the gut microbiota, inhibit necroptosis in the intestinal epithelium, increase the local Th1/Th17 response	Play role in dysbiosis and immune infiltration before disease symptoms	[40–43]
*IRGM*	Chr 5	Linkage analysis	Interact with autophagy, inflammation, and tumorigenesis	*IRGM* expression increase before the effect of bacterial autophagy, linked to UC susceptibility	[44]
*IGF2*	Chr 11	DNA from peripheral blood leucocytes and RNA from tumors of Chinese patients	The main role of IGF-2 is defined as a growth hormone in gestation	*IGF-II* LOI is present in high prevalence of CRC patients, especially, proximal cancer in Chinese population	[45]
*LIT1 (KCNQ1OT1)*	Chr 11	Colorectal cancer cell lines	Play role in transcriptional silencing of the KCNQ1	Play essential role in colorectal carcinogenesis	[46]
*PHLDA2*	Chr 11	Knockout model	Cellular apoptosis, autophagy	Downregulation of PHLDA2 inhibits tumor growth in colorectal cancer	[47]

NOD2: Nucleotide-binding oligomerization domain-containing protein 2, CARD15: Caspase recruitment domain-containing protein 15, NF-kB: Nuclear factor-kappa-B protein complex, IBD1: Inflammatory bowel disease protein 1, ATG16L1: Autophagy-related 16-like 1, IRGM: Immunity-related GTPase family M protein, UC: Ulcerative colitis, IGF2: Insulin-like growth factor II, LOI: Loss of imprinting, PHLDA2: Pleckstrin homology like domain family A member 2

#### Microbiota changes and TLRs: critical insight to IBD and colitis

For a very long time, humans and microorganisms have coexisted and developed in a mutually beneficial symbiotic relationship that is crucial for preserving homeostasis. The microbiome, however, is dynamic and adapts to aging and environmental changes. Food and dietary habits, which have changed completely in industrial societies, seem to be important environmental variables that affect the microbiota and might cause or contribute to dysbiosis. Additionally, dietary components like micronutrients have a crucial role in controlling mucosal immunity and may have a direct or indirect impact on the gut flora. Additionally, it has recently been demonstrated that some dietary ingredients might alter epigenetic processes, which may raise the risk of IBD formation and progression [48]. Through epigenetic programming of the host intestinal epithelium, the gut microbiota functions as a crucial modulator of the intestinal inflammatory response is noticeable. Microbiota activates Ten-Eleven-Translocation (TET)-dependent hypomethylation of genomic regions, which controls genes linked to inflammatory response, and is associated with colorectal cancer. The epigenetic landscape of lamina-associated domains (LADs) in colonocytes is reorganized by microbiota-induced Ten-Eleven-Translocation 3 (TET3) expression, which leads to transcriptional alterations of CRC [49]. After Lipopolysaccharides (LPS) stimulation, macrophages have an increased level of TET2, an enzyme that converts the DNA base methylcytosine to 5-hydroxymethylcytosine (5HMC). TET2-induced 5HMC has a feedback loop that prevents chronically high transcription of IL-6 during an innate immune response. In prior work, the researchers discovered that IkBζ binds TET2 to the IL-6 promoter in order to activate Histone deacetylase 2 (HDAC2) in an indirect manner, which deacetylates H3 and H4 histones and inhibits transcription [50]. Cyclooxygenases and NF-kB are two other potential contributing mechanisms. Numerous inflammation-related genes, including cyclooxygenase-2 (COX-2), nitric oxide (NO) synthase-2 (NOS)-2 and the interferon-inducible gene 1-8U, are enhanced in inflamed mucosa in UC patients. Patients with IBD have increased epithelial cell cycling in their intestinal mucosa and they have higher rates of mitosis and apoptosis than normal colonic biopsies from patients with sporadic adenomas, particularly in the active region (inflamed), as opposed to quiescent niche. Increased epithelial cell cycle, while probably contributing to carcinogenesis, is not enough to actually cause cancer [51–53]. The NF-kB and STAT3 signaling pathways, play an essential role in IBD transition to CRC and are continuously induced in cancer by numerous aberrant alterations such as epigenetics [54]. NF-kB and STAT3 signals contribute to the microenvironment carcinogenesis via inducing pro-inflammatory cytokines production, and these inflammatory mediators upregulate the expression of antiapoptotic genes, cell proliferation and angiogenesis [55]. Microbiome affects colon epithelial cells (CECs), and have remarkable effects on CECs’ non-coding RNA production [56], DNA damage [57], DNA methylation [58], and chromatin structure [59]. Genes involved in cell proliferation and the WNT signaling pathway are affected by gut microbes and play an essential role in CRC development [60].

**Fig. 2:**
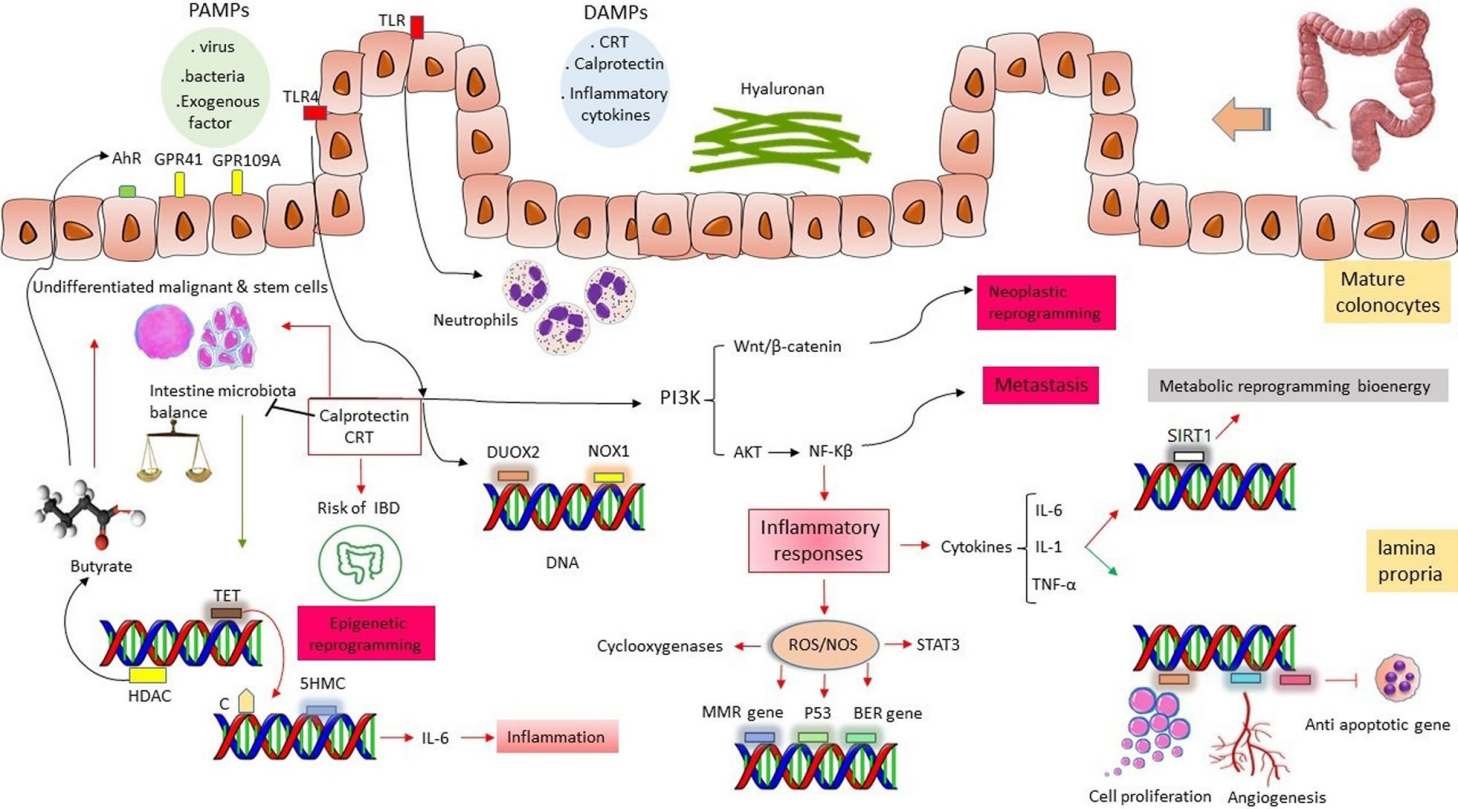
Microbiota play important role in gut environment and butyrate is an important metabolite produced by the microbiota. Microbiota induce TET expression and decrease the inflammation via TET.Butyrate inhibit HDAC, undifferentiated and stem cells, and increase the expression of AhR, GPR41, GPR109A, and mature colonocytes. PAMPs and DAMPs interact with TLRs and TLR 4 is one of the most considerable one. TLR 4 induce DUOX2 and NOX1 expression and it activates PI3K /AKT/NF-kB and PI3K/Wnt/ β-catenin signaling pathways which play important role in inflammatory response and metabolic reprogramming. PAMPs: Pathogen-associated molecular patterns, DAMPs: Damage-associated molecular patterns, TLR: Toll-like receptor, AhR: Aryl hydrocarbon receptor, GPR41: G-protein-coupled receptor 41, GPR109A: G-protein coupled receptor 109A, TET: Ten-Eleven-Translocation, HDAC: Histone deacetylase, 5HMC: 5-hydroxymethylcytosine, DUOX2: Dual oxidase 2, NOX1: NADPH Oxidase 1, STAT3: Signal transducers and activators of transcription 3, SIRT1: Sirtuin 1

Since epigenetic alterations link host gene function to environmental risk factors such as intestinal microbiota, they might have a significant impact on the etiology and the outcome of the IBD. Pathogen-associated molecular patterns (PAMPs), for example, are environmental signals that can be integrated into epigenetic processes to optimize the transcriptional production of inflammatory cytokines [61]. PAMPs activate specific Toll-Like Receptors (TLRs) in various conditions, such as inflammation and cancer [62]. Cammarota et al. indicated a putative indicator of colon cancer development is elevated TLR-4 expression in the tumor microenvironment [63]. TLR signaling has a significant impact on two key processes in wound healing: epithelial regeneration and myofibroblast activation. In the colon, a TLR2/TLR4/MyD88 cascade drives mucosal repair during the regenerative phase of colitis, demonstrating the role of TLR signals in regeneration [64, 65]. The overexpression of Dual oxidase 2 (DUOX2) and a microbiome were necessary for TLR4-dependent carcinogenesis which can be the intestinal epithelial cells respond to inflammation and ROS and oxidative stress [66]. DUOX2 and NOX1 were up-regulated in IBD and CRC as epithelial TLR4 was activated. Villin-TLR4 mice showed a large increase in epithelial hydrogen peroxide (H2O2); DUOX2 and a microbiome were critical for TLR4-dependent carcinogenesis [67]. DUOX2 contributes to innate defense against intestinal microorganisms by accelerating the production of H202 in enterocytes. An extensive Ashkenazi family with 19 Crohn disease patients hand 15 of them carry a single monoallelic exonic mutation of DUOX2, which was recently discovered. Biallelic, hereditary DUOX2 mutations were identified as a Mendelian etiology of extremely early onset IBD (VEO-IBD) in a research conducted by Parlato et al. [68]. Santaolalla et al. showed TLR4 activates β-catenin in a PI3K-dependent mechanism, increasing phosphorylation of β-catenin Ser552, a mechanism linked to activation of Wnt pathway, according to biochemical investigations in colonic epithelial cell lines. The findings imply that activation of the Wnt/β-catenin pathway by TLR4 can lead to a neoplastic reprograming [69]. Cario et al. conducted a research based on TLRs in colitis and their results TLR3 and TLR4 expression in the intestinal epithelium was differently altered in active IBD. In contrast to UC, TLR3 was markedly downregulated in the intestinal epithelial cell (IEC) of active CD. TLR4 was, however, markedly elevated in both UC and CD. In IBD, TLR2 and TLR5 expression remained unaltered. These findings show that different changes in selective TLR expression in the intestinal epithelium may be linked to IBD, and that these changes in the innate response system may play a role in the pathogenesis of these diseases [70]. TLR4-dependent PI3K/AKT/NF-κB oxidative and inflammatory pathway and gut microbiota interaction seems to have important role in intestine injury and metastasis [71]. Fusobacterium nucleatum (Fn) is a pathogen bacterium which has a poor prognosis for late stage CRC patients. Yan et al. indicated epithelial-to-mesenchymal transition (EMT) markers (E-cadherin and N-cadherin) and cancer stem cell (CSC) markers (Nanog, Oct-4, and Sox-2) are valuable markers in CRC. N-cadherin, Nanog, Oct-4, and Sox-2 were unfavorable prognostic factors, while E-cadherin was a favorable marker [72] Fig. 2.

#### Damage-associated molecular patterns (DAMPs)

Direct pro-inflammatory mediators, known as damage-associated molecular patterns (DAMPs), are endogenous stress proteins that are produced or released in response to cell or tissue damage. DAMPs might be originated from various cellular and, tissue contents, such as the nucleus, cytosol, mitochondria and extracellular matrix [73]. IBD is one of many chronic inflammatory illnesses that can be caused by excessive or persistent signaling mediated by these molecules. In IBD, the production of endogenous DAMPs such as calprotectin promotes epigenetic reprogramming and increases the inflammatory response produced by immune and non-immune cells. The outcomes influence the pathologic alterations that may maintain chronic intestinal inflammation and also cause particular disease phenotypes [74]. A 36 kDa protein belonging to the S100 family called calprotectin, has a direct antimicrobial effect, a role in the innate immune response, and is mainly generated by neutrophils. Fecal calprotectin measurement is a helpful predictor of gastrointestinal inflammation. It is a useful tool for prioritizing endoscopy since it has a strong negative predictive value for screening IBD in undiagnosed, symptomatic individuals [75]. Iron-binding lactoferrin serves as an alarming and a neutrophil degranulation indicator which might be detected in feces and used as a biomarker of IBD since it is particularly resistant to proteolysis and degradation. Consequently, lactoferrin has been used to distinguish between functional disorders and IBD [76]. The potent DAMP called calreticulin (CRT) has been found to affect homeostasis via regulating the immune system. According to recent data, CRT can translocate to the cell surface and act as a signal for immune-mediated cell death in this regard. Calreticulin plays role in anti-inflammatory programming in animal models of IBD [77]. Watanabe et al. indicated that patients with UC had significantly higher mean anti-CRT antibody titers and anti-CRT antibodies were considerably higher in these patients during the initial phase than during the passage phase. The mean anti-CRT antibody titer in CD patients was not significantly higher compared to healthy individuals [78]. A significant extracellular matrix component called hyaluronan actively contributes to IBD. In regions of inflammation, the production of this dynamic polymer is boosted and it is increased in IBD [79].

#### DNA methylation in IBD

DNA methylation in humans is the epigenetic change that has received the most attention. DNA hyper- or hypomethylation are the major way that abnormal DNA methylation leads to cancer. DNA hypomethylation is the loss of DNA methylation, which affects chromosomal stability and increases aneuploidy, whereas DNA hypermethylation causes consistent transcriptional silence and decreased gene expression. DNA methylation profile can be used as diagnostic and prognostic biomarkers in various cancers [80]. Tahara et al. suggested that EMT related genes' DNA methylation status is associated to severe clinical symptoms in UC patients [81]. Hypermethylation of vimentin (VIM) [82], OSM4 [83], SEPT9 [84], GATA4 and GATA5 [85], NDRG4 [86], BMP3 [87], ITGA4[88], plus hypomethylation of LINE1 can be used as diagnostic biomarkers for early stage detection of CRC patients [89]. Promoter hypermethylation of PRICKLE1, and other WNT pathway genes' abnormal DNA methylation contributes to the emergence and spread of CIMP-negative CRC and adenoma [90]. Hypermethylation of ITGA4, TFPI2, and VIM promoters is increased in inflamed colon tissue and it seems to be a potential risk markers for colitis-associated cancer [91]. DNA methylation profile of colon mucosa is associated to the inflammatory condition in UC patients [92] Fig. 2. Peripheral blood mononuclear cells' TFPI2 and NDRG4 gene promoter methylation analyses are new, non-invasive epigenetic biomarkers for CRC detection [93]. Based on this discussion a panel of DNA methylation profiles can be designed to estimate the IBD transition to CRC condition and it can be used in CRC diagnosis in the primary stages.

#### Histone changes in IBD

The butyric and propionic acids, which are produced by microbiota, are HDAC inhibitors, and basically, HDACs are essential for microbiota-host interaction [94]. Sodium butyrate decreases experimental colitis without IL-10 via inhibiting the NF-kB signaling pathway and histone deacetylation [95]. The modifications in H3K4me3 appeared to more accurately identify CD patients. The researchers compared these modifications to those observed in H3K4me3 in ileal epithelial cells in germ-free mice and animals kept according to standard practices. These comprehensive analyses demonstrated that the presence of gut microbiota led to several alterations in H3K4me3 in IECs [96]. Tsaprouni et al. indicated H4 was highly acetylated in inflamed biopsies and Peyer's patches (PP) of CD patients. The PP showed increased H4K5 and K16 acetylation. These findings show a link between histone acetylation and inflammation and suggest a potential new therapeutic target for mucosal inflammation [97].

#### MicroRNAs in IBD and CRC

A class of non­coding RNAs with a length of 19–25 nucleotides (nt) is known as microRNAs (miRs). They are generated by the RNaseIII family member Dicer from double-stranded hairpin precursors that are 70–100 nt in length. Dicer is a component of the RNA-induced silencing complex and is endogenously expressed in the cytoplasm. Translational repression in mammals and mRNA cleavage in plants are caused by miRs' insufficient complementarity in recognizing the 3' untranslated region of target mRNAs. miR profiling has been frequently used to identify molecular biomarkers in tumors with the emergence of high-throughput techniques like microarray and deep sequencing [98, 99]. Strong links exist between colon inflammation and miRNA dysregulation. It has been discovered that some microRNAs promote tumor progression and metastasis while others prevent tumor growth [100]. The abnormal patterns of miR expression suggest that miRs may contribute to the pathophysiology of IBD or reflect the underlying inflammation. To distinguish between CD and UC, a panel of miRs (miR-19a, miR-21, miR-31, miR-101, miR-146a, and miR-375) can be used as biomarkers [101] Table 2.

**Table 2:** miRs that can be used as a diagnostic and prognostic biomarker in IBD and CRC

*miRs*	Up/down-regulation	Diagnostic/Prognostic/ risk marker/therapeutic in disease	Role	Refernces
*miR-145, miR-143*	Down	Diagnostic/CRC High level in TNM stage I-II	Decrease cell growth in carcinoma cell line	[102]
*miR-20a, miR-143, miR-150, and let-7a*	Up	Diagnostic/CRC High level in TNM stage III	Regulate cell cycle, cell division, etc	[102]
*miR‑200c and miR-18a*	Up	Diagnostic /Prognosis/CRC	Cell proliferation, apoptosis, EMT, tumorigenesis	[103, 104]
*miR-19a*	Up	Diagnostic /CRC	Proliferation, migration, and invasion	[105]
*miR-21, miR-21-5p, miR-16-5p*	Up	Therapeutic/Prognostic/IBD-CRC	Oncogenic	[106–108]
*miR-31*	Up	Diagnosis/Prognosis/IBD-CRC	Intrinsic regulator of homeostasis, stem cell proliferation, differentiation, and epithelial cell death	[109]
*miR-155*	Up	Diagnosis/CRC	Oncogenic, immune function	[110]
*miR-214*	Up	Diagnosis/ Therapeutic/CRC	Coordinating tumor proliferation, stemness, angiogenesis, invasiveness, immune response	[111]
*miR-124*	Down	Diagnosis/CRC	Involved in the EMT, regulation of mitochondrial apoptotic pathway	[38]
*miR-193a-3p*	Down	Diagnosis/Prognosis/CRC	Proliferation, migration and invasion	[112]
*miR-139-5p*	Down	Diagnosis/Prognosis/IBD-CRC	Tumor suppressive role	[113, 114]
*miR-133a*	Down	Diagnosis, Prognosis, and Therapeutic/CRC	Proliferation, migration, and apoptosis via targeting SOX9	[115]
*miR-124a*	Down	Risk marker of Colitis-Associated Cancer in Patients with UC	Inhibit proliferation, glycolysis, and energy metabolism	[116]
*miR-155*	Down	Diagnosis/Therapeutic/IBD-CRC	Regulate immune responses	[117]
*miR-17*	Up	Diagnostic/Prognostic /IBD-CRC	Regulate cell division and cell cycle	[118]
*miR-20a*	Up	Diagnostic /CRC	Oncogenic, promoting cell growth and motility	[119]
*miR-223*	Up	Diagnostic /IBD-CRC	Differentiation and activation of granulocytes	[120]
*miR-29b*	Down	Prognostic/Therapeutic/CRC	Inhibit tumor growth and metastasis	[121]
*miR-370-3p*	Up	Prognostic/Therapeutic/ UC-CRC	Inhibiting inflammation, and EMT	[58]
*miR-31, -106a, and -135b*	Dysregulated	Prognostic/UC-CRC	Cell growth	[122]
*miR-320*	Down	Diagnostic/Prognostic/IBD-CRC	Tumor suppressor	[123]
*miR-34a/miR-34a-5p*	Down	Diagnosis/Prognosis/IBD/CRC	Inhibits tumorigenesis of CRC via c-MYC/DNMT3a/PTEN pathway	[110, 124]
*miR-146a-5p and miR-155-5p*	Up	Diagnosis, Prognosis, and Therapeutic/CRC	CRC metastasis	[125]
*miR-126*	Up	Diagnosis, Prognosis/UC	Increase inflammatory responses via NF-κB pathway	[126]

TNM: Tumor, nodes, and metastases, EMT: Epithelial–mesenchymal transition, UC: Ulcerative Colitis;

### Metabolic reprogramming in IBD and upcoming biomarkers

It is well known that long term IBD can be transitioned to CRC due to long term inflammation and imbalanced inflammatory responses [51]. Butyrate, which is mostly synthesized by bacteria, suppresses undifferentiated malignant and stem cells while stimulating mature colonocytes. Mature colonocytes use butyrate oxidation to supply 70–80% of their energy needs. In different cell types, butyrate activates the aryl hydrocarbon receptor (AhR), the GPR41, and the GPR109A receptors while inhibiting HDAC, maintaining the function of the gut barrier, and reducing inflammatory processes. The number of bacteria that produce butyrate is reduced in IBD patients. Butyrate penetrates the nucleus and acts as an inhibitor of histone deacetylase (HDACi). Cancerous colonocytes prefer glucose over butyrate as their preferred energy source because they follow the Warburg effect pathway. As a result, butyrate accumulates at moderate levels in malignant colonocytes and serves as an HDACi [127]. When TLRs identify and react to systemic life-threatening infections, gene-selective epigenetic reprogramming and modifications in cellular bioenergetics emerge. The epigenetic and bioenergy alterations are coordinated by sensor sirtuin 1 (SIRT1) Fig. 2. Liu et al. indicated SIRT1 quickly increased at the promoters of TNF-α and IL-1 after TLR4 activation, but not IκBα; SIRT1 promoter binding required its co-factor, NAD + . SIRT1 induced termination of NFκB dependent transcription by deacetylating nucleosomal histone H4 lysine 16 and RelA/p65 lysine 310 during the primary step [128]. TLR 4 is a key regulator for CRC development under high-fat diet via cancer metabolism reprogramming [129]. In metabolic diseases, the development of insulin resistance and leptin resistance is caused by the activation of TLR4 by long-chain saturated fatty acids [38]. Colon cancer cells initiate epithelial-mesenchymal transition when TLR4-mediated galectin-1 synthesis is triggered by the formation of ADAM10 and ADAM17-associated lactate [130]. Further studies indicated that the expression of glycolytic enzymes changes in chronic inflammation mainly via STAT3/c-Myc pathway, and this finding reflects metabolism reprogramming in IBD transition to CRC condition [131]. A component of the Condensin II complex, known as Non-SMC Condensin II Complex Subunit D3 (NCAPD3), is essentially responsible for chromosomal condensation and segregation during meiosis and mitosis. NCAPD3 increase glucose metabolism reprogramming and Warburg effect in CRC. The expression of NCAPD3 increase in CRC and it increase the tumorigenesis [41]. Additionally, glucose metabolism reprogramming can lead to the damage of electron transfer in mitochondria that can lead to tumorigenesis and cancer progression [132]. Hypoxia-Inducible Factor (HIF) plays essential role in CRC initiation and progression [133, 134]. HIF plays role in glucose metabolism reprogramming via increasing glucose uptake and glycolytic enzymes production [12]. Schniers et al. conducted a study on the proteome dataset of UC colon mucosa biopsies. They reported a significant difference in metallothioneins, PPAR-inducible proteins, fibrillar collagens, and proteins involved in bile acid transport as well as metabolic functions of nutrients, energy, steroids, xenobiotics, and carbonate [135]. To expand this discussion, we designed Table 3 to elucidate the importance of metabolism reprogramming which can be used as a biomarker in evaluating the condition of IBD patients. Additionally, we mentioned extra non-invasive biomarkers which can be useful in IBD and CRC management.

**Table 3:** Metabolism-relevant and non-invasive biomarkers in IBD and CRC

Biomarkers	Study	Result	Diagnostic/prognostic	References
GLUT-1	20 CRC patients	GLUT-1 overexpression associate to Tumor Size, and Depth of Invasion	Prognostic	[136]
HK1	Colonic tissue	Inflammatory role of HK-1	Can be used as a prognostic biomarker	[137]
Linoleic acid and 12-hydroxy-8,10-octadecadienoic acid	gut microbiome and the metabolic profiles were studied by using UHPLC-Q-TOF–MS/MS metabolomics and 16S rDNA sequencing technology in rats	Intestinal microbiota and host metabolism are involved in the pathophysiological process of IBD transition to CRC	Biomarkers for predicting UC transition to CRC	[138]
Serum M2-PK	Molecular epidemiology study on the serum of CRC patients	Serum M2-PK is a potential screening test for CRC	Diagnostic/early detection of CRC	[139]
Fecal tumor M2-PK	Case–control	Sensitive biomarker for pre-selection for colonoscopy	Diagnostic	[140]
Thymidine, Fumarate, Hippurate, cis-Aconitate, Pyridoxic acid, Cinnamic acid, Homogentisic acid, Indoleacetate, Trigonelline, Creatinine, Creatine, Uracil, Urea	Proton ^1^H-NMR study of CRC patients	The findings support the value of NMR-based urinary metabolomics fingerprinting for CRC early diagnosis	Diagnosis	[141]
Lipogenic enzymes, glycerophospholipids, sphingolipids, triacylglycerol	Lipidomic analysis in a cohort	CRC lipidome showed a TG-species profile	Prognostic	[142]
Six metabolic genes (NAT2, XDH, GPX3, AKR1C4, SPHK1, and ADCY5) expression	Bioinformatics analysis of Cancer Genome Atlas database	Several metabolic genes play role in the survival and clinical stage of CRC	Diagnostic/Prognostic	[143]
DMA, Arginine	CRC patients undergoing surgery	L-arginine/NO pathway metabolites can be used for screening	Prognostic	[144]
Hydroxylated, polyunsaturated ultra-long-chain fatty acids	Case–control study using FTICR-MS	These metabolites’ decrease CRC	Diagnostic	[145]
DPEP1	A meta-analysis of microarray and SAGE	DPEP1 can be used for screening of early neoplastic lesions	Prognostic	[146]
D-2-hydroxyglutarate	Mouse model	D-2-hydroxyglutarate decrease in progressive UC	Prognostic	[147]
Haemoglobin FIT	Case–control	Haemoglobin quantification in CM is a noticeable marker to detect CRC	Diagnosis	[148]
Fecal Calprotectin, and Stool Lactoferrin	Meta-analysis	FC, and SL are valuable biomarkers for defining the disease activity in IBD	Prognostic	[149]
MMP-9	A pilot study on faecal MMP-9	Fecal MMP-9 might be used as a non-invasive-biomarker	Prognostic	[150]

M2-PK: M2-pyruvate kinase, UHPLC-Q-TOF–MS/MS: Ultra-high-performance liquid chromatography combined with electrospray ionization quadrupole time-of-flight tandem mass spectrometry, H-NMR: Nuclear magnetic resonance spectroscopy, DMA: Dimethylarginines and dimethylamine, FTICR-MS : Fourier transform ion cyclotron resonance mass spectrometry, DPEP1: Dipeptidase 1, SAGE: Serial Analysis of Gene Expression, FIT: Fecal immunochemical, CM: Colorectal mucus, FC: Fecal calprotectin, SL: Stool lactoferrin, MMP-9: Matrix-metalloproteinase-9

## Conclusion and future perspectives

One of the primary challenges was to elucidate how epigenetic and metabolic reprogramming can be useful in finding suitable biomarkers for IBD and CRC management. In line with this, Epigenetics can play a crucial role in the transition of IBD to CRC. Specifically, hypermethylation of genes such as VIM, OSM4, SEPT9, GATA4, GATA5, NDRG4, BMP3, and ITGA4, along with hypomethylation of LINE1, also can potentially be utilized in the management of both IBD and CRC. Peripheral blood mononuclear cells' *TFPI2* and *NDRG4* gene promoter methylation analyses are novel, non-invasive epigenetic biomarkers which can be used as prognostic biomarkers. A panel of DNA methylation profiles can be designed to estimate the IBD transition to CRC.

Epigenetic, metabolism, and microbiome-derived biomarkers as promising non-invasive tools can be used for the accurate diagnosis and prognosis of IBD, and CRC patients. Additionally, some of these biomarkers can be used to estimate IBD transition to CRC. Linoleic acid and 12 hydroxy 8,10-octadecadienoic acid, Serum M2-pyruvate kinase and Six metabolic genes (*NAT2, XDH, GPX3, AKR1C4, SPHK1, and ADCY5*) expression are valuable biomarkers for early detection and transition to CRC condition and these biomarkers elucidate the importance of metabolism reprogramming in IBD and CRC. *miR* profiling has been frequently used to identify the link between colon inflammation and CRC. Some miRs, such as *miR-31, miR-139-5p, miR-155, miR-17, miR-223, miR-370-3p, miR-31, miR-106a, miR-135b, and miR-320* can be used as biomarkers to estimate IBD transition to CRC condition.

## Data Availability

Not applicable.

## References

[1] Sebastian-delaCruz M, Olazagoitia-Garmendia A, Gonzalez-Moro I, Santin I, Garcia-Etxebarria K, Castellanos-Rubio A (2020). Implication of m6A mRNA methylation in susceptibility to inflammatory bowel disease. Epigenomes.

[2] Kellermayer R (2012). Epigenetics and the developmental origins of inflammatory bowel diseases. Can J Gastroenterol.

[3] Ng SC, Shi HY, Hamidi N, Underwood FE, Tang W, Benchimol EI, Panaccione R, Ghosh S, Wu JC, Chan FK (2017). Worldwide incidence and prevalence of inflammatory bowel disease in the 21st century: a systematic review of population-based studies. The Lancet.

[4] Deepak P, Park SH, Ehman EC, Hansel SL, Fidler JL, Bruining DH, Fletcher JG (2017). Crohn’s disease diagnosis, treatment approach, and management paradigm: what the radiologist needs to know. Abdominal Radiol.

[5] Feuerstein JD, Cheifetz AS (2014). Ulcerative colitis: epidemiology, diagnosis, and management. Mayo Clin Proceed..

[6] Fragoulis GE, Liava C, Daoussis D, Akriviadis E, Garyfallos A, Dimitroulas T (2019). Inflammatory bowel diseases and spondyloarthropathies: from pathogenesis to treatment. World J Gastroenterol.

[7] Rogler G, Vavricka S (2015). Exposome in IBD: recent insights in environmental factors that influence the onset and course of IBD. Inflamm Bowel Dis.

[8] Kellermayer R (2017). Challenges for epigenetic research in inflammatory bowel diseases. Epigenomics.

[9] Schulmann K, Mori Y, Croog V, Yin J, Olaru A, Sterian A, Sato F, Wang S, Xu Y, Deacu E (2005). Molecular phenotype of inflammatory bowel disease-associated neoplasms with microsatellite instability. Gastroenterology.

[10] Barrat FJ, Crow MK, Ivashkiv LB (2019). Interferon target-gene expression and epigenomic signatures in health and disease. Nat Immunol.

[11] Gomase VS, Tagore S (2008). Epigenomics. Curr Drug Metab.

[12] Ghanavat M, Shahrouzian M, Zayeri ZD, Banihashemi S, Kazemi SM, Saki N (2021). Digging deeper through glucose metabolism and its regulators in cancer and metastasis. Life Sci.

[13] Ventham NT, Kennedy NA, Nimmo ER, Satsangi J (2013). Beyond gene discovery in inflammatory bowel disease: the emerging role of epigenetics. Gastroenterology.

[14] Suvà ML, Riggi N, Bernstein BE (2013). Epigenetic reprogramming in cancer. Science.

[15] Ballestar E (2011). Epigenetic alterations in autoimmune rheumatic diseases. Nat Rev Rheumatol.

[16] Sanchez-Mut JV, Gräff J (2015). Epigenetic alterations in Alzheimer’s disease. Front Behav Neurosci.

[17] Alam R, Abdolmaleky HM, Zhou JR (2017). Microbiome, inflammation, epigenetic alterations, and mental diseases. Am J Med Genet B Neuropsychiatr Genet.

[18] Ilango S, Paital B, Jayachandran P, Padma PR, Nirmaladevi R (2020). Epigenetic alterations in cancer. Front Biosci Landmark.

[19] Cleynen I, Vermeire S (2015). The genetic architecture of inflammatory bowel disease: past, present and future. Curr Opin Gastroenterol.

[20] Lange UC, Schneider R (2010). What an epigenome remembers. BioEssays.

[21] Bultman SJ (2017). Interplay between diet, gut microbiota, epigenetic events, and colorectal cancer. Mol Nutr Food Res.

[22] Niwa T, Ushijima T (2010). Induction of epigenetic alterations by chronic inflammation and its significance on carcinogenesis. Adv Genet.

[23] Mikhed Y, Görlach A, Knaus UG, Daiber A (2015). Redox regulation of genome stability by effects on gene expression, epigenetic pathways and DNA damage/repair. Redox Biol.

[24] Reeves HL, Narla G, Ogunbiyi O, Haq AI, Katz A, Benzeno S, Hod E, Harpaz N, Goldberg S, Tal-Kremer S (2004). Kruppel-like factor 6 (KLF6) is a tumor-suppressor gene frequently inactivated in colorectal cancer. Gastroenterology.

[25] Probst AV, Dunleavy E, Almouzni G (2009). Epigenetic inheritance during the cell cycle. Nat Rev Mol Cell Biol.

[26] Sen R, Barnes C (2021). Do transgenerational epigenetic inheritance and immune system development share common epigenetic processes?. J Develop Bio.

[27] Li X, Wang X, He K, Ma Y, Su N, He H, Stolc V, Tongprasit W, Jin W, Jiang J (2008). High-resolution mapping of epigenetic modifications of the rice genome uncovers interplay between DNA methylation, histone methylation, and gene expression. Plant Cell.

[28] Fiocchi C (2018). Inflammatory bowel disease: complexity and variability need integration. Front Med.

[29] Wheeler E (2022). The investigation and characterization of sex-based differences in intestinal inflammatory processes using a human organoid model. Northeastern Univ.

[30] Uhlig HH, Muise AM (2017). Clinical genomics in inflammatory bowel disease. Trends Genet.

[31] Brooks J. Exploring Genetic Susceptibility: Using a combined systems biology, in vitro and ex vivo approach to understand the pathology of ulcerative colitis. University of East Anglia. 2018.

[32] Lawson HA, Cheverud JM, Wolf JB (2013). Genomic imprinting and parent-of-origin effects on complex traits. Nat Rev Genet.

[33] Fu J, Zhang L, Li D, Tian T, Wang X, Sun H, Ge A, Liu Y, Zhang X, Huang H (2022). DNA Methylation of Imprinted Genes KCNQ1, KCNQ1OT1, and PHLDA2 in peripheral blood is associated with the risk of breast cancer. Cancers.

[34] Jima DD, Skaar DA, Planchart A, Motsinger-Reif A, Cevik SE, Park SS, Cowley M, Wright F, House J, Liu A (2022). Genomic map of candidate human imprint control regions: the imprintome. Epigenetics.

[35] Gao T, Liu X, He B, Pan Y, Wang S (2020). IGF2 loss of imprinting enhances colorectal cancer stem cells pluripotency by promoting tumor autophagy. Aging.

[36] O'Neill H. Transitioning from analysing methylation profiles in bulk populations of Colorectal cancer cells to methylation profiles of single cells. University of Otago. 2021.

[37] Sakatani T, Kaneda A, Iacobuzio-Donahue CA, Carter MG, de Boom WS, Okano H, Ko MS, Ohlsson R, Longo DL, Feinberg AP (2005). Loss of imprinting of Igf2 alters intestinal maturation and tumorigenesis in mice. Science.

[38] Shahmohamadnejad S, Nouri Ghonbalani Z, Tahbazlahafi B, Panahi G, Meshkani R, Emami Razavi A, Shokri Afra H, Khalili E (2020). Aberrant methylation of miR-124 upregulates DNMT3B in colorectal cancer to accelerate invasion and migration. Archiv Physiol Biochem.

[39] Goethel A, Turpin W, Rouquier S, Zanello G, Robertson S, Streutker C, Philpott D, Croitoru K (2019). Nod2 influences microbial resilience and susceptibility to colitis following antibiotic exposure. Mucosal Immunol.

[40] Matsuzawa-Ishimoto Y, Shono Y, Gomez LE, Hubbard-Lucey VM, Cammer M, Neil J, Dewan MZ, Lieberman SR, Lazrak A, Marinis JM (2017). Autophagy protein ATG16L1 prevents necroptosis in the intestinal epithelium. J Exp Med.

[41] Jing Z, Liu Q, He X, Jia Z, Xu Z, Yang B, Liu P (2022). NCAPD3 enhances Warburg effect through c-myc and E2F1 and promotes the occurrence and progression of colorectal cancer. J Exp Clin Cancer Res.

[42] Lavoie S, Conway KL, Lassen KG, Jijon HB, Pan H, Chun E, Michaud M, Lang JK, Comeau CAG, Dreyfuss JM (2019). The Crohn’s disease polymorphism, ATG16L1 T300A, alters the gut microbiota and enhances the local Th1/Th17 response. Elife.

[43] Cader MZ, Kaser A (2013). Recent advances in inflammatory bowel disease: mucosal immune cells in intestinal inflammation. Gut.

[44] Sharma A, Szymczak S, Rühlemann M, Freitag-Wolf S, Knecht C, Enderle J, Schreiber S, Franke A, Lieb W, Krawczak M (2022). Linkage analysis identifies novel genetic modifiers of microbiome traits in families with inflammatory bowel disease. Gut microbes.

[45] Liou J-M, Wu M-S, Lin J-T, Wang H-P, Huang S-P, Chiu H-M, Lee Y-C, Lin Y-B, Shun C-T, Liang J-T (2007). Loss of imprinting of insulin-like growth factor II is associated with increased risk of proximal colon cancer. Eur J Cancer.

[46] Nakano S, Murakami K, Meguro M, Soejima H, Higashimoto K, Urano T, Kugoh H, Mukai T, Ikeguchi M, Oshimura M (2006). Expression profile of LIT1/KCNQ1OT1 and epigenetic status at the KvDMR1 in colorectal cancers. Cancer Sci.

[47] Ma Z, Lou S, Jiang Z (2020). PHLDA2 regulates EMT and autophagy in colorectal cancer via the PI3K/AKT signaling pathway. Aging (Albany NY).

[48] Rapozo DC, Bernardazzi C, de Souza HSP (2017). Diet and microbiota in inflammatory bowel disease: the gut in disharmony. World J Gastroenterol.

[49] Zouggar A, Haebe JR, Benoit YD (2020). Intestinal microbiota influences DNA methylome and susceptibility to colorectal cancer. Genes.

[50] Hu Y, Yan F, Ying L, Xu D (2017). Emerging roles for epigenetic programming in the control of inflammatory signaling integration in heath and disease. Regulat Inflammat Signal Health Dis..

[51] Itzkowitz SH, Yio X (2004). Inflammation and cancer IV. Colorectal cancer in inflammatory bowel disease: the role of inflammation. Am J Physiol Gastrointest Liver Physiol.

[52] Ieda T, Tazawa H, Okabayashi H, Yano S, Shigeyasu K, Kuroda S, Ohara T, Noma K, Kishimoto H, Nishizaki M (2019). Visualization of epithelial-mesenchymal transition in an inflammatory microenvironment–colorectal cancer network. Sci Rep.

[53] Wang Y, Wang P, Shao L (2021). Correlation of ulcerative colitis and colorectal cancer: a systematic review and meta-analysis. J Gastroint Oncol.

[54] Patel M, Horgan PG, McMillan DC, Edwards J (2018). NF-κB pathways in the development and progression of colorectal cancer. Transl Res.

[55] Luo C, Zhang H (2017). The role of proinflammatory pathways in the pathogenesis of colitis-associated colorectal cancer. Med Inflammat.

[56] Abi Zamer B, Abumustafa W, Hamad M, Maghazachi AA, Muhammad JS (2021). Genetic mutations and non-coding RNA-based epigenetic alterations mediating the warburg effect in colorectal carcinogenesis. Biology.

[57] González-Sánchez P, DeNicola GM (2021). The microbiome (s) and cancer: know thy neighbor (s). J Pathol.

[58] Lin L, Wang D, Qu S, Zhao H, Lin Y (2020). miR-370-3p alleviates ulcerative colitis-related colorectal cancer in mice through inhibiting the inflammatory response and epithelial-mesenchymal transition. Drug Des Dev Ther.

[59] Ryu TY, Kim K, Han T-S, Lee M-O, Lee J, Choi J, Jung KB, Jeong E-J, An DM, Jung C-R (2022). Human gut-microbiome-derived propionate coordinates proteasomal degradation via HECTD2 upregulation to target EHMT2 in colorectal cancer. ISME J.

[60] Liu W, Crott JW, Lyu L, Pfalzer AC, Li J, Choi S-W, Yang Y, Mason JB, Liu Z (2016). Diet-and genetically-induced obesity produces alterations in the microbiome, inflammation and Wnt pathway in the intestine of Apc+/1638N mice: comparisons and contrasts. J Cancer.

[61] Nanini HF, Bernardazzi C, Castro F, de Souza HSP (2018). Damage-associated molecular patterns in inflammatory bowel disease: From biomarkers to therapeutic targets. World J Gastroenterol.

[62] Miggin SM, O’neill LA (2006). New insights into the regulation of TLR signaling. J Leukoc Biol.

[63] Cammarota R, Bertolini V, Pennesi G, Bucci EO, Gottardi O, Garlanda C, Laghi L, Barberis MC, Sessa F, Noonan DM (2010). The tumor microenvironment of colorectal cancer: stromal TLR-4 expression as a potential prognostic marker. J Transl Med.

[64] Deravi N, Poudineh M, Pirzadeh M, Yavarpour-Bali H, Mehrabi H, Erabi G, Saghazadeh A, Rezaei N (2022). The Yin and Yang of toll-like receptors in endothelial dysfunction. Int Immunopharmacol.

[65] Kluwe J, Mencin A, Schwabe RF (2009). Toll-like receptors, wound healing, and carcinogenesis. J Mol Med.

[66] Burgueño JF, Fritsch J, Santander AM, Brito N, Fernández I, Pignac-Kobinger J, Conner GE, Abreu MT (2019). Intestinal epithelial cells respond to chronic inflammation and dysbiosis by synthesizing H2O2. Front Physiol.

[67] Burgueño JF, Fritsch J, González EE, Landau KS, Santander AM, Fernández I, Hazime H, Davies JM, Santaolalla R, Phillips MC (2021). Epithelial TLR4 signaling activates DUOX2 to induce microbiota-driven tumorigenesis. Gastroenterology.

[68] Parlato M, Charbit-Henrion F, Hayes P, Tiberti A, Aloi M, Cucchiara S, Bègue B, Bras M, Pouliet A, Rakotobe S (2017). First identification of biallelic inherited DUOX2 inactivating mutations as a cause of very early onset inflammatory bowel disease. Gastroenterology.

[69] Santaolalla R, Sussman DA, Ruiz JR, Davies JM, Pastorini C, Espana CL, Sotolongo J, Burlingame O, Bejarano PA, Philip S (2013). TLR4 activates the β-catenin pathway to cause intestinal neoplasia. PLoS ONE.

[70] Cario E, Podolsky DK (2000). Differential alteration in intestinal epithelial cell expression of toll-like receptor 3 (TLR3) and TLR4 in inflammatory bowel disease. Infect Immun.

[71] Fu Q, Song T, Ma X, Cui J (2022). Research progress on the relationship between intestinal microecology and intestinal bowel disease. Animal Models Exp Med..

[72] Yan X, Liu L, Li H, Qin H, Sun Z (2017). Clinical significance of Fusobacterium nucleatum, epithelial–mesenchymal transition, and cancer stem cell markers in stage III/IV colorectal cancer patients. Onco Targets Ther.

[73] Krysko DV, Garg AD, Kaczmarek A, Krysko O, Agostinis P, Vandenabeele P (2012). Immunogenic cell death and DAMPs in cancer therapy. Nat Rev Cancer.

[74] Foell D, Wittkowski H, Roth J (2009). Monitoring disease activity by stool analyses: from occult blood to molecular markers of intestinal inflammation and damage. Gut.

[75] Walsham NE, Sherwood RA (2016). Fecal calprotectin in inflammatory bowel disease. Clin Exp Gastroenterol.

[76] Wang Y, Pei F, Wang X, Sun Z, Hu C, Dou H (2015). Diagnostic accuracy of fecal lactoferrin for inflammatory bowel disease: a meta-analysis. Int J Clin Exp Pathol.

[77] Ohkuro M, Kim J-D, Kuboi Y, Hayashi Y, Mizukami H, Kobayashi-Kuramochi H, Muramoto K, Shirato M, Michikawa-Tanaka F, Moriya J (2018). Calreticulin and integrin alpha dissociation induces anti-inflammatory programming in animal models of inflammatory bowel disease. Nat Commun.

[78] Watanabe K, Ohira H, Orikasa H, Saito K, Kanno K, Shioya Y, Obara K, Sato Y (2006). Anti-calreticulin antibodies in patients with inflammatory bowel disease. Fukushima J Med Sci.

[79] Tomasz J, Andrzej B (2022). Hyaluronan reduces colitis-induced intraperitoneal inflammation during peritoneal dialysis. Perit Dial Int.

[80] Rasras S, Zibara K, Vosughi T, Zayeri Z (2018). Genetics and epigenetics of glioblastoma: therapeutic challenges. Clin Cancer Invest J.

[81] Tahara T, Shibata T, Okubo M, Ishizuka T, Nakamura M, Nagasaka M, Nakagawa Y, Ohmiya N, Arisawa T, Hirata I (2014). DNA methylation status of epithelial-mesenchymal transition (EMT)-related genes is associated with severe clinical phenotypes in ulcerative colitis (UC). PLoS ONE.

[82] Shirahata A, Hibi K (2014). Serum vimentin methylation as a potential marker for colorectal cancer. Anticancer Res.

[83] Anto EM, Nair A, Purushothaman J (2021). Emerging role of circulating tumour DNA in treatment response prognosis in colon cancer. Colon Cancer Diagnosis Therapy..

[84] Wang Y, Chen P-M, Liu R-B (2018). Advance in plasma SEPT9 gene methylation assay for colorectal cancer early detection. World J Gastrointest Oncol.

[85] Lu H, Huang S, Zhang X, Wang D, Zhang X, Yuan X, Zhang Q, Huang Z (2014). DNA methylation analysis of SFRP2, GATA4/5, NDRG4 and VIM for the detection of colorectal cancer in fecal DNA. Oncol Lett.

[86] Xiao W, Zhao H, Dong W, Li Q, Zhu J, Li G, Zhang S, Ye M (2015). Quantitative detection of methylated NDRG4 gene as a candidate biomarker for diagnosis of colorectal cancer. Oncol Lett.

[87] Rokni P, Shariatpanahi AM, Sakhinia E, Kerachian MA (2018). BMP3 promoter hypermethylation in plasma-derived cell-free DNA in colorectal cancer patients. Genes Genomics.

[88] Jafarpour S, Saberi F, Yazdi M, Nedaeinia R, Amini G, Ferns GA, Salehi R (2022). Association between colorectal cancer and the degree of ITGA4 promoter methylation in peripheral blood mononuclear cells. Gene Reports.

[89] Kerachian MA, Kerachian M (2019). Long interspersed nucleotide element-1 (LINE-1) methylation in colorectal cancer. Clin Chim Acta.

[90] Gul S, Wenru T (2022). Highlight DNA methylation biomarkers in different cancer type for drug designing. J Epigenet.

[91] Gerecke C, Scholtka B, Löwenstein Y, Fait I, Gottschalk U, Rogoll D, Melcher R, Kleuser B (2015). Hypermethylation of ITGA4, TFPI2 and VIMENTIN promoters is increased in inflamed colon tissue: putative risk markers for colitis-associated cancer. J Cancer Res Clin Oncol.

[92] Hartnett L, Egan LJ (2012). Inflammation, DNA methylation and colitis-associated cancer. Carcinogenesis.

[93] Bagheri H, Mosallaei M, Bagherpour B, Khosravi S, Salehi AR, Salehi R (2020). TFPI2 and NDRG4 gene promoter methylation analysis in peripheral blood mononuclear cells are novel epigenetic noninvasive biomarkers for colorectal cancer diagnosis. J Gene Med.

[94] Mirzaei R, Dehkhodaie E, Bouzari B, Rahimi M, Gholestani A, Hosseini-Fard SR, Keyvani H, Teimoori A, Karampoor S (2022). Dual role of microbiota-derived short-chain fatty acids on host and pathogen. Biomed Pharmacother.

[95] Lee C, Kim BG, Kim JH, Chun J, Im JP, Kim JS (2017). Sodium butyrate inhibits the NF-kappa B signaling pathway and histone deacetylation, and attenuates experimental colitis in an IL-10 independent manner. Int Immunopharmacol.

[96] Kelly D, Kotliar M, Woo V, Jagannathan S, Whitt J, Moncivaiz J, Aronow BJ, Dubinsky MC, Hyams JS, Markowitz JF (2018). Microbiota-sensitive epigenetic signature predicts inflammation in Crohn’s disease. JCI insight.

[97] Tsaprouni LG, Ito K, Powell JJ, Adcock IM, Punchard N (2011). Differential patterns of histone acetylation in inflammatory bowel diseases. J Inflamm.

[98] Kim VN, Han J, Siomi MC (2009). Biogenesis of small RNAs in animals. Nat Rev Mol Cell Biol.

[99] Wei J-W, Huang K, Yang C, Kang C-S (2017). Non-coding RNAs as regulators in epigenetics. Oncol Rep.

[100] Kalla R, Ventham N, Kennedy N, Quintana J, Nimmo E, Buck A, Satsangi J (2015). MicroRNAs: new players in IBD. Gut.

[101] Schaefer JS, Attumi T, Opekun AR, Abraham B, Hou J, Shelby H, Graham DY, Streckfus C, Klein JR (2015). MicroRNA signatures differentiate Crohn’s disease from ulcerative colitis. BMC Immunol.

[102] Maminezhad H, Ghanadian S, Pakravan K, Razmara E, Rouhollah F, Mossahebi-Mohammadi M, Babashah S (2020). A panel of six-circulating miRNA signature in serum and its potential diagnostic value in colorectal cancer. Life Sci.

[103] Zhang G-J, Zhou T, Liu Z-L, Tian H-P, Xia S-S (2013). Plasma miR-200c and miR-18a as potential biomarkers for the detection of colorectal carcinoma. Mol Clin Oncol.

[104] Shen K, Cao Z, Zhu R, You L, Zhang T (2019). The dual functional role of MicroRNA-18a (miR-18a) in cancer development. Clin Transl Med.

[105] Li H, Huang B (2022). miR-19a targets CLCA4 to regulate the proliferation, migration, and invasion of colorectal cancer cells. Eur J Histochem.

[106] Shi C, Yang Y, Xia Y, Okugawa Y, Yang J, Liang Y, Chen H, Zhang P, Wang F, Han H (2016). Novel evidence for an oncogenic role of microRNA-21 in colitis-associated colorectal cancer. Gut.

[107] Okugawa Y, Yao L, Toiyama Y, Yamamoto A, Shigemori T, Yin C, Omura Y, Ide S, Kitajima T, Shimura T (2018). Prognostic impact of sarcopenia and its correlation with circulating miR-21 in colorectal cancer patients. Oncol Rep.

[108] Zhou R, Qiu P, Wang H, Yang H, Yang X, Ye M, Wang F, Zhao Q (2021). Identification of microRNA-16-5p and microRNA-21-5p in feces as potential noninvasive biomarkers for inflammatory bowel disease. Aging.

[109] Olaru AV, Selaru FM, Mori Y, Vazquez C, David S, Paun B, Cheng Y, Jin Z, Yang J, Agarwal R (2011). Dynamic changes in the expression of MicroRNA-31 during inflammatory bowel disease-associated neoplastic transformation. Inflamm Bowel Dis.

[110] Zhao J, Lin H, Huang K (2022). Mesenchymal stem cell-derived extracellular vesicles transmitting microRNA-34a-5p suppress tumorigenesis of colorectal cancer through c-MYC/DNMT3a/PTEN axis. Mol Neurobiol.

[111] Sharma T, Hamilton R, Mandal CC (2015). miR-214: a potential biomarker and therapeutic for different cancers. Future Oncol.

[112] Ying H, Lin F, Ding R, Wang W, Hong W (2020). Extracellular vesicles carrying miR-193a derived from mesenchymal stem cells impede cell proliferation, migration and invasion of colon cancer by downregulating FAK. Exp Cell Res.

[113] Grillo TG, Quaglio AEV, Beraldo RF, Lima TB, Baima JP, Di Stasi LC, Sassaki LY (2021). MicroRNA expression in inflammatory bowel disease-associated colorectal cancer. World J Gastrointestinal Oncol.

[114] Zhang L, Dong Y, Zhu N, Tsoi H, Zhao Z, Wu CW, Wang K, Zheng S, Ng SS, Chan FK (2014). microRNA-139-5p exerts tumor suppressor function by targeting NOTCH1 in colorectal cancer. Mol Cancer.

[115] Lamichhane S, Mo J-S, Sharma G, Joung S-M, Chae S-C (2022). MIR133A regulates cell proliferation, migration, and apoptosis by targeting SOX9 in human colorectal cancer cells. Am J Cancer Res.

[116] Ueda Y, Ando T, Nanjo S, Ushijima T, Sugiyama T (2014). DNA methylation of microRNA-124a is a potential risk marker of colitis-associated cancer in patients with ulcerative colitis. Dig Dis Sci.

[117] Liu J, Chen Z, Xiang J, Gu X (2018). MicroRNA-155 acts as a tumor suppressor in colorectal cancer by targeting CTHRC1 in vitro. Oncol Lett.

[118] Lai H, Zhang J, Zuo H, Liu H, Xu J, Feng Y, Lin Y, Mo X (2020). Overexpression of miR-17 is correlated with liver metastasis in colorectal cancer. Medicine.

[119] Yau TO, Wu CW, Tang C-M, Chen Y, Fang J, Dong Y, Liang Q, Ng SSM, Chan FKL, Sung JJY (2016). MicroRNA-20a in human faeces as a non-invasive biomarker for colorectal cancer. Oncotarget.

[120] James JP, Riis LB, Malham M, Høgdall E, Langholz E, Nielsen BS (2020). MicroRNA biomarkers in IBD—differential diagnosis and prediction of Colitis-Associated cancer. Int J Mol Sci.

[121] Wang B, Li W, Liu H, Yang L, Liao Q, Cui S, Wang H, Zhao L (2014). miR-29b suppresses tumor growth and metastasis in colorectal cancer via downregulating Tiam1 expression and inhibiting epithelial–mesenchymal transition. Cell Death Dis.

[122] Quintanilla I, Jung G, Jimeno M, Lozano JJ, Sidorova J, Camps J, Carballal S, Bujanda L, Vera MI, Quintero E (2022). Differentially deregulated microRNAs as novel biomarkers for neoplastic progression in ulcerative colitis. Clin Transl Gastroenterol.

[123] Wu M-Y, Luo Y-X, Jia W-X, Wang D-D, Sun D-L, Song J, Wang J, Niu W-W, Zhang X-L (2022). miRNA-320 inhibits colitis-associated colorectal cancer by regulating the IL-6R/STAT3 pathway in mice. J Gastrointestinal Oncol.

[124] Fouad A, Tarek M, Abdel Hamid RA, Mahmoud YH, Mohamed AA, Saleh M, Samir N (2022). Serum miR-34a as a potential biomarker for diagnosis of inflammatory bowel diseases in Egyptian patients. Egyptian J Int Med.

[125] Wang D, Wang X, Song Y, Si M, Sun Y, Liu X, Cui S, Qu X, Yu X (2022). Exosomal miR-146a-5p and miR-155-5p promote CXCL12/CXCR7-induced metastasis of colorectal cancer by crosstalk with cancer-associated fibroblasts. Cell Death Dis.

[126] Yu C, Zhang G, Ye S, Tian T, Liang Q, Cui L, Cen J, Hu J, Zheng R, Wang H (2022). Regulatory mechanisms of miRNA-126 expression in ulcerative colitis. Int J Mol Sci.

[127] Gasaly N, Hermoso MA, Gotteland M (2021). Butyrate and the fine-tuning of colonic homeostasis: implication for inflammatory bowel diseases. Int J Mol Sci.

[128] Liu TF, Yoza BK, El Gazzar M, Vachharajani VT, McCall CE (2011). NAD+-dependent SIRT1 deacetylase participates in epigenetic reprogramming during endotoxin tolerance. J Biol Chem.

[129] Tong Y, Gao H, Qi Q, Liu X, Li J, Gao J, Li P, Wang Y, Du L, Wang C (2021). High fat diet, gut microbiome and gastrointestinal cancer. Theranostics.

[130] Sun L, Chen B, Wu J, Jiang C, Fan Z, Feng Y, Xu Y (2020). Epigenetic regulation of a Disintegrin and metalloproteinase (ADAM) transcription in colorectal cancer cells: Involvement of β-catenin, BRG1, and KDM4. Front Cell Develop Bio.

[131] Qu D, Shen L, Liu S, Li H, Ma Y, Zhang R, Wu K, Yao L, Li J, Zhang J (2017). Chronic inflammation confers to the metabolic reprogramming associated with tumorigenesis of colorectal cancer. Cancer Biol Ther.

[132] Hart P, Mao M, de Abreu A, Ansenberger-Fricano K, Ekoue D, Ganini D, Kajdacsy-Balla A, Diamond A, Minshall R, Consolaro M (2015). MnSOD upregulation sustains the Warburg effect via mitochondrial ROS and AMPK-dependent signalling in cancer. Nat Commun.

[133] Eda S, Vadde R, Jinka R (2017). Role of hypoxia-inducible factor (HIF) in the initiation of cancer and its therapeutic inhibitors. Role Transcript Factors Gastrointestinal Malignanc.

[134] Xue X, Taylor M, Anderson E, Hao C, Qu A, Greenson JK, Zimmermann EM, Gonzalez FJ, Shah YM (2012). Hypoxia-inducible factor-2α activation promotes colorectal cancer progression by dysregulating iron homeostasis. Can Res.

[135] Schniers A, Goll R, Pasing Y, Sørbye SW, Florholmen J, Hansen T (2019). Ulcerative colitis: functional analysis of the in-depth proteome. Clin Proteomics.

[136] Gu J, Yamamoto H, Fukunaga H, Danno K, Takemasa I, Ikeda M, Tatsumi M, Sekimoto M, Hatazawa J, Nishimura T (2006). Correlation of GLUT-1 overexpression, tumor size, and depth of invasion with 18F-2-fluoro-2-deoxy-D-glucose uptake by positron emission tomography in colorectal cancer. Dig Dis Sci.

[137] Dai L, Perera DS, King DW, Southwell BR, Burcher E, Liu L (2012). Hemokinin-1 stimulates prostaglandin E2 production in human colon through activation of cyclooxygenase-2 and inhibition of 15-hydroxyprostaglandin dehydrogenase. J Pharmacol Exp Ther.

[138] Tang Q, Cang S, Jiao J, Rong W, Xu H, Bi K, Li Q, Liu R (2020). Integrated study of metabolomics and gut metabolic activity from ulcerative colitis to colorectal cancer: the combined action of disordered gut microbiota and linoleic acid metabolic pathway might fuel cancer. J Chromatogr A.

[139] Meng W, Zhu H-H, Xu Z-F, Cai S-R, Dong Q, Pan Q-R, Zheng S, Zhang S-Z (2012). Serum M2-pyruvate kinase: a promising non-invasive biomarker for colorectal cancer mass screening. World J Gastrointestinal Oncol.

[140] Tonus C, Neupert G, Sellinger M (2006). Colorectal cancer screening by non-invasive metabolic biomarker fecal tumor M2-PK. World J Gastroenterol.

[141] Wang Z, Lin Y, Liang J, Huang Y, Ma C, Liu X, Yang J (2017). NMR-based metabolomic techniques identify potential urinary biomarkers for early colorectal cancer detection. Oncotarget.

[142] Ecker J, Benedetti E, Kindt AS, Höring M, Perl M, Machmüller AC, Sichler A, Plagge J, Wang Y, Zeissig S (2021). The colorectal cancer lipidome: identification of a robust tumor-specific lipid species signature. Gastroenterology.

[143] Miao Y, Li Q, Wang J, Quan W, Li C, Yang Y, Mi D (2020). Prognostic implications of metabolism-associated gene signatures in colorectal cancer. PeerJ.

[144] Bednarz-Misa I, Fleszar MG, Zawadzki M, Kapturkiewicz B, Kubiak A, Neubauer K, Witkiewicz W, Krzystek-Korpacka M (2020). L-Arginine/NO pathway metabolites in colorectal cancer: relevance as disease biomarkers and predictors of adverse clinical outcomes following surgery. J Clin Med.

[145] Ritchie SA, Ahiahonu PW, Jayasinghe D, Heath D, Liu J, Lu Y, Jin W, Kavianpour A, Yamazaki Y, Khan AM (2010). Reduced levels of hydroxylated, polyunsaturated ultra long-chain fatty acids in the serum of colorectal cancer patients: implications for early screening and detection. BMC Med.

[146] Eisenach P, Soeth E, Röder C, Klöppel G, Tepel J, Kalthoff H, Sipos B (2013). Dipeptidase 1 (DPEP1) is a marker for the transition from low-grade to high-grade intraepithelial neoplasia and an adverse prognostic factor in colorectal cancer. Br J Cancer.

[147] Han J, Jackson D, Holm J, Turner K, Ashcraft P, Wang X, Cook B, Arning E, Genta RM, Venuprasad K (2018). Elevated d-2-hydroxyglutarate during colitis drives progression to colorectal cancer. Proc Natl Acad Sci.

[148] Loktionov A, Soubieres A, Bandaletova T, Francis N, Allison J, Sturt J, Mathur J, Poullis A (2020). Biomarker measurement in non-invasively sampled colorectal mucus as a novel approach to colorectal cancer detection: screening and triage implications. Br J Cancer.

[149] Mosli MH, Zou G, Garg SK, Feagan SG, MacDonald JK, Chande N, Sandborn WJ, Feagan BG (2015). C-reactive protein, fecal calprotectin, and stool lactoferrin for detection of endoscopic activity in symptomatic inflammatory bowel disease patients: a systematic review and meta-analysis. Official J Am College Gastroenterol ACG.

[150] Annaházi A, Ábrahám S, Farkas K, Rosztóczy A, Inczefi O, Földesi I, Szűcs M, Rutka M, Theodorou V, Eutamene H (2016). A pilot study on faecal MMP-9: a new noninvasive diagnostic marker of colorectal cancer. Br J Cancer.

